# One-dimensional ladder gallium coordination polymer

**DOI:** 10.1107/S2056989019013446

**Published:** 2019-10-03

**Authors:** Andrea B. Simões, Flávio Figueira, Ricardo F. Mendes, Jéssica S. Barbosa, João Rocha, Filipe A. Almeida Paz

**Affiliations:** a CICECO–Aveiro Institute of Materials, Department of Chemistry, University of Aveiro, Campus Universitário de Santiago, 3810-193 Aveiro, Portugal; b QOPNA & LAQV-REQUIMTE, Chemistry Department, University of Aveiro, 3810-193 Aveiro, Portugal

**Keywords:** crystal structure, coordination polymers, gallium

## Abstract

A one-dimensional ladder-type coordination polymer, poly[[(μ_2_-hydroxido)(μ_2_-1*H*-pyrazole-3,5-di­carboxyl­ato)gallium(III)] monohydrate], [Ga(C_5_H_2_N_2_O_4_)(OH)(H_2_O)]_*n*_ or [Ga(HPDC)(OH)(H_2_O)]_*n*_, **I**, isotypic with a V^3+^ coordination polymer previously reported by Chen *et al.* [*J. Coord. Chem.* (2008). **61**, 3556–3567] was prepared from Ga^3+^ and pyrazole-3,5-di­carb­oxy­lic acid monohydrate (H_3_PDC·H_2_O). Compound **I** was isolated using three distinct experimental methods: hydro­thermal (HT), microwave-assisted (MWAS) and one-pot (OP) and the crystallite size should be fine-tuned according to the method employed. The coordination polymeric structure is based on a dimeric Ga^3+^ moiety comprising two μ_2_-bridging hydroxide groups, which are inter­connected by HPDC^2−^ anionic organic linkers. The close packing of individual polymers is strongly directed by the supra­molecular inter­actions, namely several O—H⋯O and N—H⋯O hydrogen-bonding inter­actions.

## Abstract 2

The one-dimensional [Ga(HPDC)(OH)(H_2_O)]_*n*_ ladder-type coordination polymer can be prepared using three distinct synthetic approaches (hydro­thermal, microwave-assisted and a one-pot process) with crystallite size varying according to the method employed.

## Chemical Context   

Research on coordination polymers (CPs) and metal–organic frameworks (MOFs) remains a topical area in chemistry, particularly the study of their crystal structures (Cui *et al.*, 2016 ▸). These crystalline materials are typically obtained from a combination of metal ions and organic mol­ecules, giving rise to one-, two- or three-dimensional structures (Chaplais *et al.*, 2009 ▸). A wide variety of synthetic methods have been reported for the preparation of CPs/MOFs (Stock & Biswas, 2012 ▸; Yuan *et al.*, 2018 ▸) ranging from ambient-temperature synthesis to conventional [hydro­thermal (HT) and one-pot processes (OP)] and microwave (MWAS) synthesis. In addition, other less common techniques such as electrochemistry (EC), mechanochemistry (MC) and ultrasonic (US) synthesis can be used (Rubio-Martinez *et al.*, 2017 ▸; Stock & Biswas, 2012 ▸).

A large number of MOF-containing divalent transition-metal ions have been described (Stock & Biswas, 2012 ▸; Devic & Serre, 2014 ▸). Examples of CPs/MOFs containing main-group elements, such as Al^3+^, Ga^3+^, or In^3+^, remain scarce (Stock, 2014 ▸). A search in the Cambridge Structural Database unveils around 100 Ga^3+^-bearing CP/MOF structures, for example. Remarkably, most of these structures were solved using powder X-ray diffraction (PXRD) techniques (Reinsch & De Vos, 2014 ▸; Volkringer *et al.*, 2009 ▸). Such materials exhibit high thermal and chemical stability and are ideal candidates for a wide variety of applications (Silva *et al.*, 2015 ▸; Yuan *et al.*, 2018 ▸; Ajoyan *et al.*, 2018 ▸; Howarth *et al.*, 2016 ▸). As a result of the close similarity of the coordination chemistry of gallium and aluminium, most of the Ga^3+^-CP/MOFs published are isotypic with well-known Al^3+^-CP/MOFs, and also with the much rarer In^3+^-CP/MOFs (Schilling *et al.*, 2016 ▸). This may, in part, explain why most of the studies found in the literature of Ga^3+^-CP/MOFs report only their structures. Furthermore, most of the applications that have been studied are related to those that are also found for Al^3+^-CP/MOFs (Banerjee *et al.*, 2011 ▸; Zhang *et al.*, 2018 ▸; Reinsch & De Vos, 2014 ▸; Canivet *et al.*, 2014 ▸; Zhou *et al.*, 2012 ▸). For certain applications, Ga^3+^-CP/MOFs excel, even surpassing the performance of the Al^3+^-CP/MOFs (Coudert *et al.*, 2014 ▸; Ramaswamy *et al.*, 2017 ▸; Weber *et al.*, 2016 ▸; Gao *et al.*, 2014 ▸). Furthermore, gallium materials possess low toxicity and are found in applications such as gas and water adsorption, shock-absorber technology and semiconductors (Ramaswamy *et al.*, 2017 ▸; Coudert *et al.*, 2014 ▸; Schilling *et al.*, 2016 ▸)

Following our inter­est in CP/MOFs, we have attempted the preparation of MOF-303 (Fathieh *et al.*, 2018 ▸) with Ga^3+^. In this crystallographic report we describe these studies, which culminated in the isolation of a compact one-dimensional ladder coordination polymer, [Ga(HPDC)(OH)(H_2_O)] (**I**), prepared by the self-assembly of Ga^3+^ and the organic linker 3,5-pyrazoledi­carb­oxy­lic acid monohydrate (H_3_PDC·H_2_O). Compound **I** was obtained using a variety of methods (hydro­thermal, microwave and a one-pot process) and a survey of the literature revealed that it is isotypic with a compound published in 2008 (Chen *et al.*, 2008 ▸), which is not unprecedented (Volkringer *et al.*, 2009 ▸; Finsy *et al.*, 2009 ▸; Volkringer *et al.*, 2008 ▸).
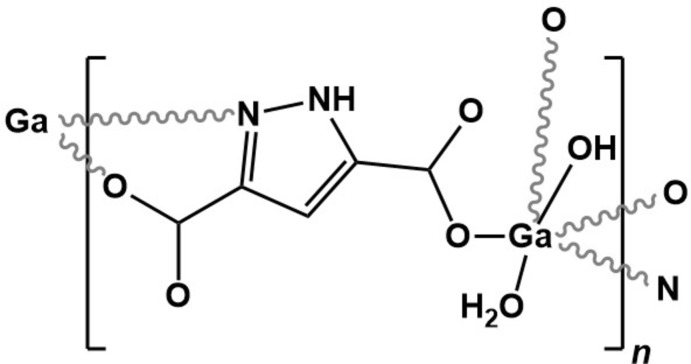


## Crystal Morphology and Characterization   

Compound **I** was prepared by hydro­thermal (HT), microwave (MWAS) and one-pot (OP) synthesis. The general experimental conditions were similar (solvent, molar qu­anti­ties and temperature). The compound is isotypic with [V(HPDC)(OH)(H_2_O)] (Chen *et al.*, 2008 ▸), which was prepared using harsher conditions. Our attempts to obtain the analogous V^3+^-bearing material using the conditions described here were unsuccessful.

**Figure 1:**
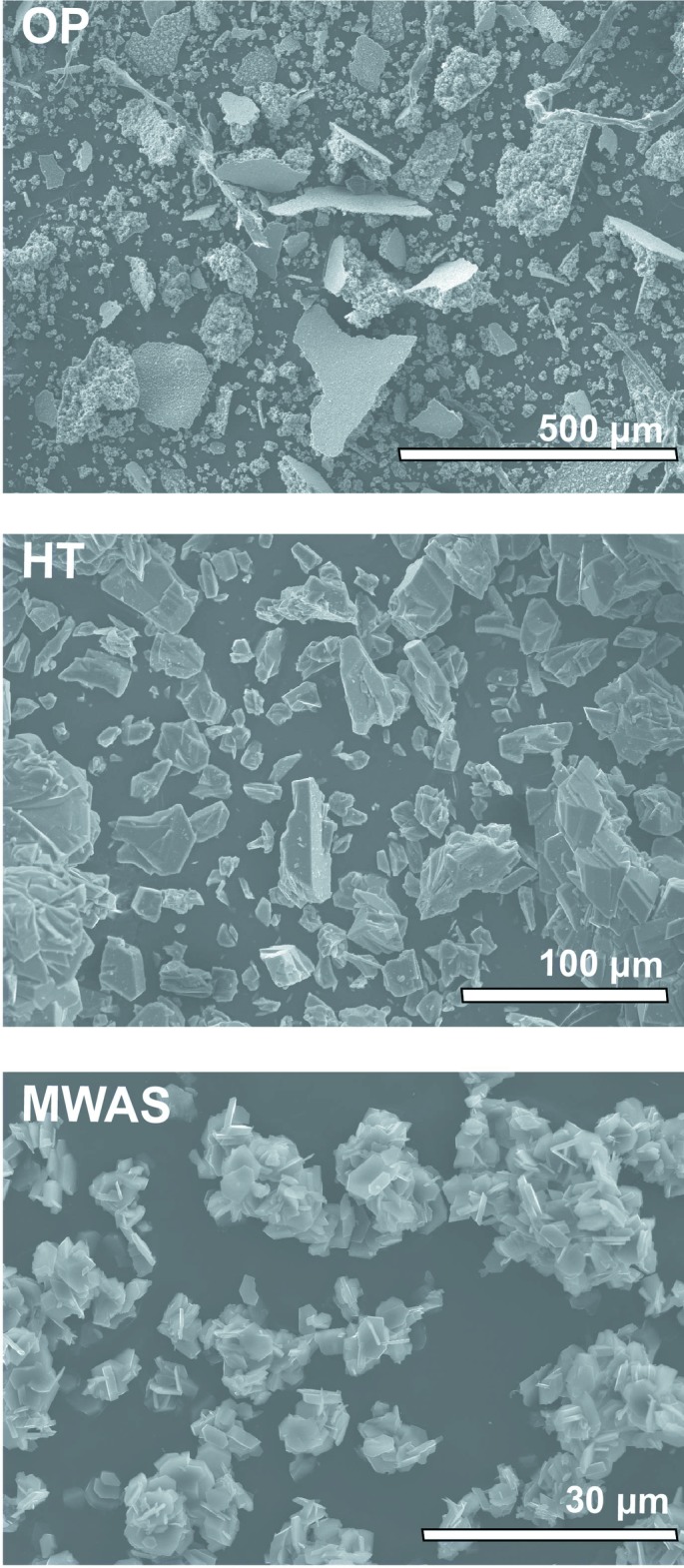
Scanning electron microscopy (SEM) images of bulk [Ga(HPDC)(OH)(H_2_O)] (**I**) obtained by microwave-assisted synthesis (MWAS), hydro­thermal synthesis (HT) and a one-pot process (OP).

MWAS produces phase pure coordination polymers much faster than the HT and OP approaches. PXRD studies have confirmed the same structural features (see Figure S1 in the supporting information). The crystal morphology, however, varied depending on the method employed (Fig. 1 ▸). Crystals typically exhibit irregular shapes. MWAS allowed a faster preparation of **I** when compared to the other methods (a reduction from 24 h to just 1 h) with a significantly smaller average crystal size (*ca* 3–5 µm) and a more uniform plate-like morphology. The HT method, on the other hand, afforded larger crystals (*ca* 15–65 µm) with a more block-type morphology, while the OP method resulted mainly in agglomerated particles with a plate-like morphology.

FT–IR spectroscopy supports the structural features revealed by the X-ray diffraction studies (Figure S2 in the supporting information). Compound **I** exhibits two broad bands centred at 3280 and 3159 cm^−1^ attributed to the O—H stretching vibrational modes from the coordinated water mol­ecules and to the N—H stretching vibrations of the pyrazole ring. In the central region of the spectrum, between *ca* 1700 and 1300 cm^−1^, it is possible to discern the typical C—O, C—C and C—N stretching vibrational modes arising from the pyrazole rings and the bending vibration of water mol­ecules.

The materials showed similar thermal decomposition profiles between ambient temperature and *ca* 1000 K (Figure S3 in the supporting information). Between ambient temperature and *ca* 548 K, there is almost no weight loss, which is indicative of good thermal stability. The weight loss registered between *ca* 548 and 618 K is 14.9, 14.6 and 14.8% for the OP-, HT- and MWAS-derived materials, respectively, and is attributed to the release of the water of coordination and the decomposition of the hydroxyl group (theoretical weight loss 14.0%). The subsequent weight loss (*ca* 44.9%) is attributed to the decomposition of the ligand, resulting in the formation of Ga_2_O_3_.

## Structural Commentary   

**Figure 2:**
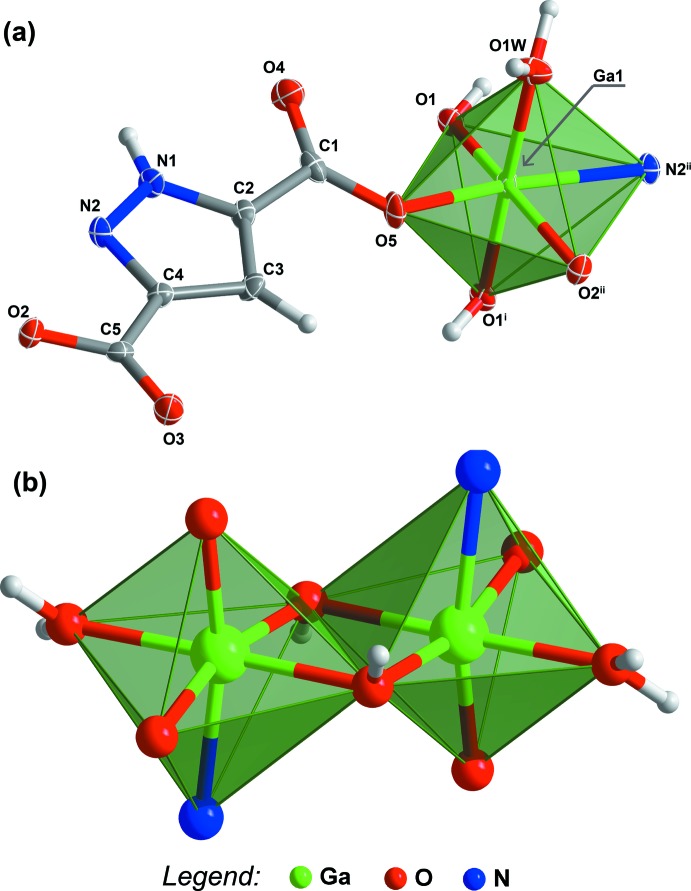
(*a*) Schematic representation of the asymmetric unit of [Ga(HPDC)(OH)(H_2_O)] (**I**) showing all non-H atoms shown with displacement ellipsoids drawn at the 50% probability level and H atoms as small spheres with arbitrary radii. The coordination sphere of the crystallographically independent metal centre was completed by generating the remaining atoms through symmetry. [Symmetry codes: (i) −*x*, −*y*, −*z* + 2; (ii) *x*, *y*, *z* + 1 (*b*) Ga^3+^ dimer formed by two symmetry-related bridging hydroxyl groups.

Compound **I** was formulated by single-crystal X-ray diffraction as [Ga(HPDC)(OH)(H_2_O)] from a crystal obtained using hydro­thermal synthetic conditions (see *Experimental* section for further details). This compound crystallizes in the centrosymmetric *P* 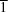 space group with the asymmetric unit being composed of one Ga^3+^ metal centre, one HPDC^2−^ anionic organic linker, one hydroxyl group and one coordin­ated water mol­ecule, as depicted in Fig. 2 ▸ *a*.

The anionic organic linker HPDC^2−^ has two distinct coord­ination modes: forming a *N*,*O*-chelate with the crystallographically independent Ga^3+^ metal centre [bite angle of 78.02 (11)°], and bridging with an adjacent metal centre through a *syn* inter­action involving the carboxyl­ate group, imposing a Ga⋯Ga inter­metallic distance of *ca* 8.52 Å (*i.e*. the length of the *c* axis of the unit cell). The octa­hedral {GaNO_5_} coordination sphere is completed by one water mol­ecule and two-symmetry related μ_2_-bridging hydroxyl groups, which are the responsible for the formation of a centrosymmetric dimer, as depicted in Fig. 2 ▸ *b* (inter­metallic distance of *ca* 2.97 Å).

The Ga—O bond lengths range between 1.903 (3) and 1.988 (3) Å and the Ga—N distance is 2.112 (3) Å (Table 1 ▸), in good agreement with those reported for other carboxyl­ate-based materials as witnessed by a search in the Cambridge Structural Database (CSD version of 2019; Groom *et al.*, 2016 ▸): mean value of 1.988 Å for the Ga—O bond (CSD range, 1.832–2.475 Å) and 2.023 Å for the Ga—N bond (CSD range 1.798–3.275 Å).

**Figure 3:**
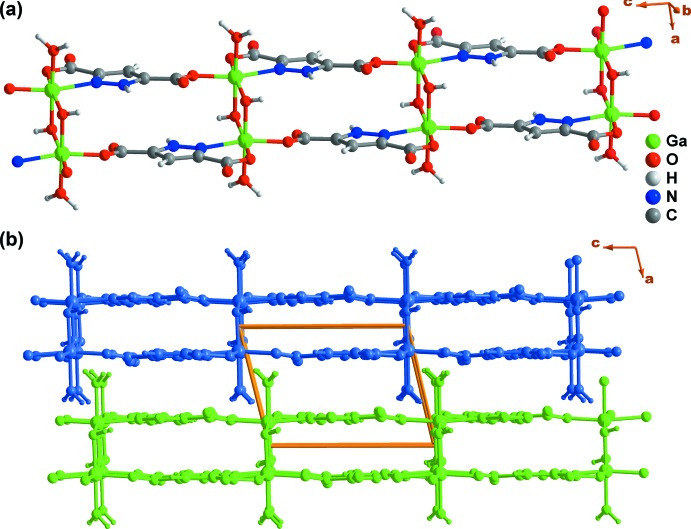
Schematic representation of the (*a*) one-dimensional ladder-type coordination polymer present in **I**, and (*b*) the close packing of the polymers viewed along the [010] direction of the unit cell.

The aforementioned connectivity promotes the formation of a one-dimensional ladder-type coordination polymer along the [001] direction (Fig. 3 ▸ *a*), which close pack in a parallel fashion in the *ab* plane of the unit cell mediated by various supra­molecular contacts (see the following section). Although the organic linkers are stacked within these ladders, the inter-centroid distance is 4.442 (3) Å, indicating the absence of significant π–π supra­molecular inter­actions.

## Supra­molecular Features   

**Figure 4:**
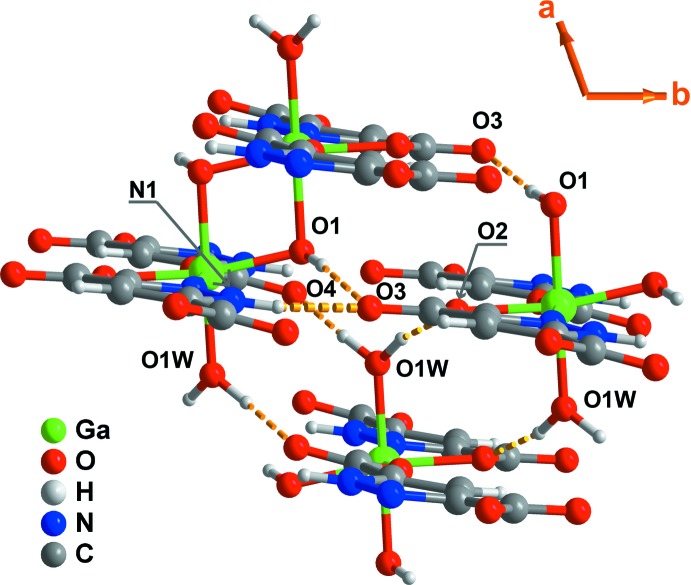
Schematic representation of a portion of the crystal packing of [Ga(HPDC)(OH)(H_2_O)] (**I**) depicting the O—H⋯O and N—H⋯O supra­molecular contacts (orange dashed lines) between ladder-type polymers. For geometrical details on the represented inter­actions see Table 2 ▸ (the symmetry codes used to generate equivalent atoms have been omitted for clarity).

Compound **I** contains several functional groups that can promote the formation of various hydrogen-bonding inter­actions (Fig. 4 ▸, Table 2 ▸). The coordinated water mol­ecule is engaged in two strong and directional O—H⋯O hydrogen-bonding inter­actions with neighbouring carboxyl­ate groups from adjacent one-dimensional chains: *d_D_*_⋯*A*_ distances of 2.643 (4) and 2.803 (4) Å and <(*D*H*A*) angles in the 164–165° range (Fig. 4 ▸, Table 2 ▸). These inter­actions may be described by the graph set motifs, 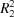
(12) and 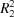
(20). The independent μ_2_-bridging hydroxyl group also donates a hydrogen atom to a neighbouring carbonyl group (from the *N*,*O*-chelated moiety) in a strong inter­action: *d_D_*_⋯*A*_ distance of 2.751 (4) Å and <(*D*H*A*) angle of 175°. This contact leads to the formation of a supra­molecular chain 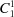
(10) across neighbouring polymers. Like the μ_2_-bridging hydroxyl group, the pyrazole ring is involved in a N—H⋯O inter­action with a *N*,*O*-chelated ligand, leading to the formation of a distinct type of supra­molecular chain, 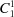
(6) [*d_D_*_⋯*A*_ distance of 2.773 (4) Å and <(*D*H*A*) angle of 152°].

**Figure 5:**
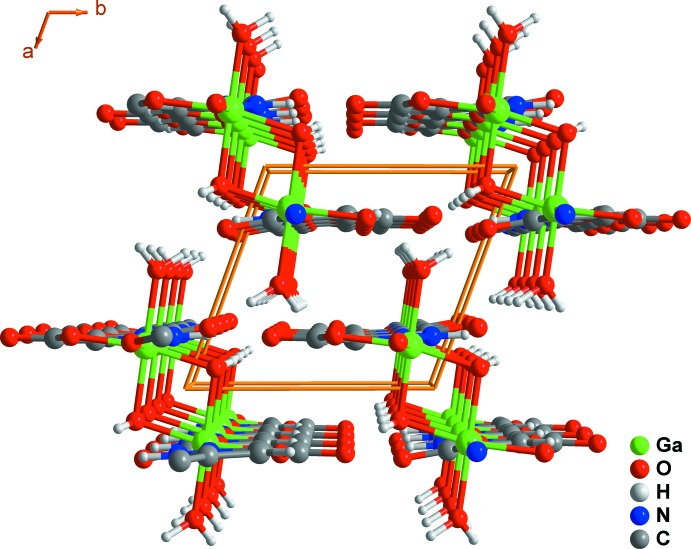
Crystal packing of [Ga(HPDC)(OH)(H_2_O)] (**I**) viewed along the [001] direction of the unit cell.

These supra­molecular inter­actions lead to a close packing of individual polymers and to a compact crystal structure of **I**, as shown in Fig. 5 ▸.

## Synthesis and Crystallization Procedures   

Chemicals were purchased from commercial sources (Merck and TCI) and used without any further purification. The methods and molar qu­anti­ties described here were based on a methodology described by Yaghi and coworkers for the preparation of MOF-303 (Fathieh *et al.*, 2018 ▸).

Compound **I** was prepared by dissolving 147 mg (0.57 mmol) of gallium nitrate hexa­hydrate (Ga(NO_3_)_3_·6H_2_O) and 100 mg (0.57 mmol) of 3,5-pyrazoledi­carb­oxy­lic acid monohydrate (H_3_PDC·H_2_O) in 9.6 mL of water in the corres­ponding vessel (autoclave for hydro­thermal synthesis, microwave vial for microwave synthesis and a round-bottom flask equipped with a condenser for the one-pot approach). Subsequently, the vessels were heated at 373 K for 24 h (hydro­thermal and one-pot synthesis) or 1 h (microwave synthesis). The resulting white precipitates were separated by filtration, washed three times with water and three times with ethanol, and dried overnight at ambient temperature (yields: 49, 47 and 60% for the one-pot, hydro­thermal and microwave-assisted syntheses, respectively).

## Refinement   

Crystal data, data collection and structure refinement details are summarized in Table 3 ▸. Hydrogen atoms bound to carbon and nitro­gen were placed at idealized positions using the HFIX 43 instruction in *SHELXL2018/*3 and included in subsequent refinement with C—H = 0.95 Å and N—H = 0.88 Å with the isotropic thermal displacement parameters fixed at 1.2*U*_eq_ of the atom to which they are attached.

Hydrogen atoms from the coordinated water mol­ecule and the hydroxyl group were directly located from difference-Fourier maps and included in the final structural models with the O—H and H⋯H distances restrained to 0.95 (1) and 1.55 (1) Å, respectively, in order to ensure a chemically reasonable environment. These hydrogen atoms were modelled with the isotropic thermal displacement parameters fixed at 1.5*U*_eq_(O).

## Supplementary Material

Crystal structure: contains datablock(s) I. DOI: 10.1107/S2056989019013446/sj5578sup1.cif

Structure factors: contains datablock(s) I. DOI: 10.1107/S2056989019013446/sj5578Isup2.hkl

Supporting Information. DOI: 10.1107/S2056989019013446/sj5578sup3.docx

CCDC references: 1956892, 1956892

Additional supporting information:  crystallographic information; 3D view; checkCIF report

## Figures and Tables

**Table 1:** Selected geometric parameters (Å, °)

Ga1—O1	1.903 (3)	Ga1—O2^ii^	1.987 (3)
Ga1—O5	1.932 (3)	Ga1—O1*W*	1.988 (3)
Ga1—O1^i^	1.974 (3)	Ga1—N2^ii^	2.112 (3)
			
O1—Ga1—O5	101.59 (12)	O1^i^—Ga1—O1*W*	178.65 (12)
O1—Ga1—O1^i^	79.94 (13)	O2^ii^—Ga1—O1*W*	91.00 (11)
O5—Ga1—O1^i^	93.13 (12)	O1—Ga1—N2^ii^	93.94 (12)
O1—Ga1—O2^ii^	166.31 (11)	O5—Ga1—N2^ii^	164.18 (12)
O5—Ga1—O2^ii^	87.28 (11)	O1^i^—Ga1—N2^ii^	92.49 (12)
O1^i^—Ga1—O2^ii^	89.26 (11)	O2^ii^—Ga1—N2^ii^	78.02 (11)
O1—Ga1—O1*W*	99.60 (12)	O1*W*—Ga1—N2^ii^	86.27 (12)
O5—Ga1—O1*W*	88.20 (12)		

Symmetry codes: (i) 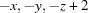; (ii) 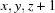.

**Table 2:** Hydrogen-bond geometry (Å, °)

*D*—H⋯*A*	*D*—H	H⋯*A*	*D*⋯*A*	*D*—H⋯*A*
O1*W*—H1*X*⋯O4^iii^	0.94	1.73	2.643 (4)	164
O1*W*—H1*Y*⋯O2^iv^	0.94	1.89	2.803 (4)	165
O1—H1⋯O3^v^	0.77	1.99	2.751 (4)	175
N1—H1*N*⋯O3^vi^	0.88	1.96	2.773 (4)	152

Symmetry codes: (iii) 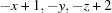; (iv) 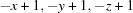; (v) 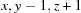; (vi) 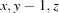.

**Table 3:** Experimental details

Crystal data	
Chemical formula	[Ga(C_5_H_2_N_2_O_4_)(OH)(H_2_O)]
*M*_r_	258.83
Crystal system, space group	Triclinic, *P* 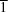
Temperature (K)	150
*a*, *b*, *c* (Å)	6.6055 (13), 6.8830 (16), 8.5178 (19)
α, β, γ (°)	94.804 (8), 101.306 (7), 108.596 (7)
*V* (Å^3^)	355.44 (14)
*Z*	2
Radiation type	Mo *K*α
μ (mm^−1^)	3.88
Crystal size (mm)	0.13 × 0.10 × 0.07

Data collection	
Diffractometer	Bruker D8 QUEST
Absorption correction	Multi-scan (*SADABS*; Bruker, 2001 ▸; Krause *et al.* 2015 ▸)
No. of measured, independent and observed [*I* > 2σ(*I*)] reflections	5768, 1303, 1182
*R*_int_	0.034
(sin θ/λ)_max_ (Å^−1^)	0.603

Refinement	
*R*[*F* ^2^ > 2σ(*F* ^2^)], *wR*(*F* ^2^), *S*	0.031, 0.074, 1.11
No. of reflections	1303
No. of parameters	136
No. of restraints	3
H-atom treatment	H atoms treated by a mixture of independent and constrained refinement
Δρ_max_, Δρ_min_ (e Å^−3^)	0.65, −0.47

Computer programs: *APEX3* (Bruker, 2016 ▸), *SAINT* (Bruker, 2015 ▸), *SHELXT2014* (Sheldrick, 2015*a* ▸), *SHELXL2018* (Sheldrick, 2015*b* ▸), *PLATON* (Spek, 2009 ▸) and *DIAMOND* (Brandenburg, 1999 ▸).

## supplementary crystallographic information

### Crystal data

**Table:**

[Ga(C_5_H_2_N_2_O_4_)(OH)(H_2_O)]	*Z* = 2
*M**_r_* = 258.83	*F*(000) = 256
Triclinic, *P*1	*D*_x_ = 2.418 Mg m^−^^3^
*a* = 6.6055 (13) Å	Mo *K*α radiation, λ = 0.71073 Å
*b* = 6.8830 (16) Å	Cell parameters from 3255 reflections
*c* = 8.5178 (19) Å	θ = 2.5–25.4°
α = 94.804 (8)°	µ = 3.88 mm^−^^1^
β = 101.306 (7)°	*T* = 150 K
γ = 108.596 (7)°	Block, colourless
*V* = 355.44 (14) Å^3^	0.13 × 0.10 × 0.07 mm

### Data collection

**Table:**

Bruker D8 QUEST diffractometer	1303 independent reflections
Radiation source: Sealed tube	1182 reflections with *I* > 2σ(*I*)
Multi-layer X-ray mirror monochromator	*R*_int_ = 0.034
Detector resolution: 10.4167 pixels mm^-1^	θ_max_ = 25.4°, θ_min_ = 3.7°
ω / φ scans	*h* = −7→7
Absorption correction: multi-scan (SADABS; Bruker, 2001; Krause *et al.* 2015)	*k* = −8→8
	*l* = −10→10
5768 measured reflections	

### Refinement

**Table:**

Refinement on *F*^2^	3 restraints
Least-squares matrix: full	Hydrogen site location: mixed
*R*[*F*^2^ > 2σ(*F*^2^)] = 0.031	H atoms treated by a mixture of independent and constrained refinement
*wR*(*F*^2^) = 0.074	*w* = 1/[σ^2^(*F*_o_^2^) + (0.0292*P*)^2^ + 1.2151*P*] where *P* = (*F*_o_^2^ + 2*F*_c_^2^)/3
*S* = 1.11	(Δ/σ)_max_ = 0.001
1303 reflections	Δρ_max_ = 0.65 e Å^−^^3^
136 parameters	Δρ_min_ = −0.47 e Å^−^^3^

### Special details

**Table:**

Geometry. All esds (except the esd in the dihedral angle between two l.s. planes) are estimated using the full covariance matrix. The cell esds are taken into account individually in the estimation of esds in distances, angles and torsion angles; correlations between esds in cell parameters are only used when they are defined by crystal symmetry. An approximate (isotropic) treatment of cell esds is used for estimating esds involving l.s. planes.

### Fractional atomic coordinates and isotropic or equivalent isotropic displacement parameters (Å^2^)

**Table:**

	*x*	*y*	*z*	*U*_iso_*/*U*_eq_	
Ga1	0.20874 (7)	0.17985 (7)	1.03804 (5)	0.00844 (15)	
O1W	0.5321 (4)	0.2428 (4)	1.0908 (3)	0.0140 (6)	
H1X	0.604 (6)	0.162 (6)	1.147 (5)	0.021*	
H1Y	0.627 (5)	0.335 (5)	1.040 (5)	0.021*	
O1	0.1126 (4)	−0.1152 (4)	1.0094 (3)	0.0100 (6)	
H1	0.142 (8)	−0.167 (7)	1.082 (6)	0.015*	
N1	0.2437 (5)	0.1027 (5)	0.4128 (4)	0.0102 (7)	
H1N	0.249943	−0.023469	0.404671	0.012*	
N2	0.2363 (5)	0.2116 (5)	0.2909 (4)	0.0088 (7)	
C1	0.2537 (6)	0.1216 (6)	0.7048 (5)	0.0103 (8)	
C2	0.2405 (6)	0.2131 (6)	0.5511 (4)	0.0089 (8)	
C3	0.2296 (6)	0.4004 (6)	0.5154 (5)	0.0102 (8)	
H3	0.223752	0.511146	0.586837	0.012*	
C4	0.2289 (6)	0.3927 (6)	0.3507 (4)	0.0074 (8)	
C5	0.2388 (6)	0.5450 (6)	0.2360 (5)	0.0087 (8)	
O2	0.2500 (4)	0.4783 (4)	0.0937 (3)	0.0098 (6)	
O3	0.2423 (5)	0.7207 (4)	0.2790 (3)	0.0137 (6)	
O4	0.2886 (5)	−0.0428 (4)	0.7039 (3)	0.0149 (6)	
O5	0.2257 (4)	0.2323 (4)	0.8208 (3)	0.0123 (6)	

### Atomic displacement parameters (Å^2^)

**Table:**

	*U*^11^	*U*^22^	*U*^33^	*U*^12^	*U*^13^	*U*^23^
Ga1	0.0123 (2)	0.0088 (2)	0.0059 (2)	0.00441 (17)	0.00399 (16)	0.00291 (15)
O1W	0.0120 (14)	0.0172 (16)	0.0176 (15)	0.0074 (13)	0.0071 (12)	0.0109 (12)
O1	0.0142 (14)	0.0097 (14)	0.0078 (14)	0.0050 (12)	0.0032 (11)	0.0044 (11)
N1	0.0164 (17)	0.0109 (16)	0.0069 (16)	0.0069 (14)	0.0057 (13)	0.0056 (13)
N2	0.0110 (17)	0.0093 (16)	0.0061 (15)	0.0025 (13)	0.0034 (13)	0.0029 (12)
C1	0.0076 (19)	0.014 (2)	0.0075 (19)	0.0009 (16)	0.0010 (15)	0.0034 (15)
C2	0.0073 (19)	0.012 (2)	0.0071 (19)	0.0018 (16)	0.0035 (15)	0.0016 (15)
C3	0.011 (2)	0.0117 (19)	0.0100 (19)	0.0053 (16)	0.0060 (16)	0.0018 (15)
C4	0.0066 (18)	0.0077 (18)	0.0084 (18)	0.0020 (15)	0.0034 (14)	0.0024 (15)
C5	0.0071 (19)	0.0087 (19)	0.0112 (19)	0.0040 (15)	0.0015 (15)	0.0018 (15)
O2	0.0158 (14)	0.0064 (13)	0.0086 (13)	0.0043 (11)	0.0048 (11)	0.0022 (10)
O3	0.0205 (16)	0.0120 (15)	0.0110 (14)	0.0084 (12)	0.0036 (12)	0.0033 (11)
O4	0.0203 (16)	0.0171 (16)	0.0125 (14)	0.0100 (13)	0.0076 (12)	0.0072 (12)
O5	0.0182 (15)	0.0139 (14)	0.0052 (13)	0.0050 (12)	0.0036 (11)	0.0028 (11)

### Geometric parameters (Å, º)

**Table:**

Ga1—O1	1.903 (3)	N1—H1N	0.8800
Ga1—O5	1.932 (3)	N2—C4	1.326 (5)
Ga1—O1^i^	1.974 (3)	C1—O4	1.224 (5)
Ga1—O2^ii^	1.987 (3)	C1—O5	1.276 (5)
Ga1—O1W	1.988 (3)	C1—C2	1.499 (5)
Ga1—N2^ii^	2.112 (3)	C2—C3	1.369 (6)
Ga1—Ga1^i^	2.9716 (10)	C3—C4	1.399 (5)
O1W—H1X	0.943 (10)	C3—H3	0.9500
O1W—H1Y	0.939 (10)	C4—C5	1.487 (5)
O1—H1	0.77 (5)	C5—O3	1.225 (5)
N1—N2	1.333 (4)	C5—O2	1.284 (5)
N1—C2	1.355 (5)		
			
O1—Ga1—O5	101.59 (12)	Ga1—O1—H1	118 (4)
O1—Ga1—O1^i^	79.94 (13)	Ga1^i^—O1—H1	107 (4)
O5—Ga1—O1^i^	93.13 (12)	N2—N1—C2	110.6 (3)
O1—Ga1—O2^ii^	166.31 (11)	N2—N1—H1N	124.7
O5—Ga1—O2^ii^	87.28 (11)	C2—N1—H1N	124.7
O1^i^—Ga1—O2^ii^	89.26 (11)	C4—N2—N1	106.8 (3)
O1—Ga1—O1W	99.60 (12)	C4—N2—Ga1^iii^	112.3 (2)
O5—Ga1—O1W	88.20 (12)	N1—N2—Ga1^iii^	140.7 (3)
O1^i^—Ga1—O1W	178.65 (12)	O4—C1—O5	129.1 (4)
O2^ii^—Ga1—O1W	91.00 (11)	O4—C1—C2	118.2 (3)
O1—Ga1—N2^ii^	93.94 (12)	O5—C1—C2	112.7 (3)
O5—Ga1—N2^ii^	164.18 (12)	N1—C2—C3	107.5 (3)
O1^i^—Ga1—N2^ii^	92.49 (12)	N1—C2—C1	119.5 (3)
O2^ii^—Ga1—N2^ii^	78.02 (11)	C3—C2—C1	133.0 (4)
O1W—Ga1—N2^ii^	86.27 (12)	C2—C3—C4	104.8 (3)
O1—Ga1—Ga1^i^	40.86 (8)	C2—C3—H3	127.6
O5—Ga1—Ga1^i^	99.50 (8)	C4—C3—H3	127.6
O1^i^—Ga1—Ga1^i^	39.08 (8)	N2—C4—C3	110.3 (3)
O2^ii^—Ga1—Ga1^i^	127.84 (8)	N2—C4—C5	115.1 (3)
O1W—Ga1—Ga1^i^	140.45 (9)	C3—C4—C5	134.4 (3)
N2^ii^—Ga1—Ga1^i^	94.18 (9)	O3—C5—O2	124.3 (4)
Ga1—O1W—H1X	124 (2)	O3—C5—C4	121.2 (3)
Ga1—O1W—H1Y	123 (2)	O2—C5—C4	114.5 (3)
H1X—O1W—H1Y	110.8 (16)	C5—O2—Ga1^iii^	118.6 (2)
Ga1—O1—Ga1^i^	100.06 (13)	C1—O5—Ga1	129.9 (3)
			
C2—N1—N2—C4	0.2 (4)	N1—N2—C4—C5	175.1 (3)
C2—N1—N2—Ga1^iii^	−173.6 (3)	Ga1^iii^—N2—C4—C5	−9.2 (4)
N2—N1—C2—C3	0.2 (4)	C2—C3—C4—N2	0.7 (4)
N2—N1—C2—C1	−178.4 (3)	C2—C3—C4—C5	−173.7 (4)
O4—C1—C2—N1	7.0 (5)	N2—C4—C5—O3	−177.0 (3)
O5—C1—C2—N1	−173.1 (3)	C3—C4—C5—O3	−2.8 (7)
O4—C1—C2—C3	−171.2 (4)	N2—C4—C5—O2	1.0 (5)
O5—C1—C2—C3	8.7 (6)	C3—C4—C5—O2	175.2 (4)
N1—C2—C3—C4	−0.5 (4)	O3—C5—O2—Ga1^iii^	−173.3 (3)
C1—C2—C3—C4	177.8 (4)	C4—C5—O2—Ga1^iii^	8.8 (4)
N1—N2—C4—C3	−0.5 (4)	O4—C1—O5—Ga1	−4.2 (6)
Ga1^iii^—N2—C4—C3	175.2 (2)	C2—C1—O5—Ga1	175.8 (2)

Symmetry codes: (i) −*x*, −*y*, −*z*+2; (ii) *x*, *y*, *z*+1; (iii) *x*, *y*, *z*−1.

### Hydrogen-bond geometry (Å, º)

**Table:**

*D*—H···*A*	*D*—H	H···*A*	*D*···*A*	*D*—H···*A*
O1*W*—H1*X*···O4^iv^	0.94	1.73	2.643 (4)	164
O1*W*—H1*Y*···O2^v^	0.94	1.89	2.803 (4)	165
O1—H1···O3^vi^	0.77	1.99	2.751 (4)	175
N1—H1*N*···O3^vii^	0.88	1.96	2.773 (4)	152

Symmetry codes: (iv) −*x*+1, −*y*, −*z*+2; (v) −*x*+1, −*y*+1, −*z*+1; (vi) *x*, *y*−1, *z*+1; (vii) *x*, *y*−1, *z*.

## References

[bb1] Ajoyan, Z., Marino, P. & Howarth, A. J. (2018). *CrystEngComm*, **20**, 5899–5912.

[bb2] Banerjee, D., Kim, S. J., Wu, H., Xu, W., Borkowski, L. A., Li, J. & Parise, J. B. (2011). *Inorg. Chem.* **50**, 208–212. 10.1021/ic101789u 21141851

[bb3] Brandenburg, K. (1999). *DIAMOND*. Crystal Impact GbR, Bonn, Germany.

[bb4] Bruker (2001). *SADABS*. Bruker AXS Inc., Madison, Wisconsin, USA.

[bb5] Bruker (2015). *SAINT*. Bruker AXS Inc., Madison, Wisconsin, USA.

[bb6] Bruker (2016). *APEX3*. Bruker AXS Inc., Madison, Wisconsin, USA.

[bb7] Canivet, J., Bonnefoy, J., Daniel, C., Legrand, A., Coasne, B. & Farrusseng, D. (2014). *New J. Chem.* **38**, 3102–3111.

[bb8] Chaplais, G., Simon-Masseron, A., Porcher, F., Lecomte, C., Bazer-Bachi, D., Bats, N. & Patarin, J. (2009). *Phys. Chem. Chem. Phys.* **11**, 5241–5245. 10.1039/b822163d 19551190

[bb9] Chen, H., Ma, C., Xiang, S., Hu, M., Si, Y., Chen, C. & Liu, Q. (2008). *J. Coord. Chem.* **61**, 3556–3567.

[bb10] Coudert, F.-X., Ortiz, A. U., Haigis, V., Bousquet, D., Fuchs, A. H., Ballandras, A., Weber, G., Bezverkhyy, I., Geoffroy, N., Bellat, J.-P., Ortiz, G., Chaplais, G., Patarin, J. & Boutin, A. (2014). *J. Phys. Chem. C*, **118**, 5397–5405.

[bb11] Cui, Y., Li, B., He, H., Zhou, W., Chen, B. & Qian, G. (2016). *Acc. Chem. Res.* **49**, 483–493. 10.1021/acs.accounts.5b00530 26878085

[bb12] Devic, T. & Serre, C. (2014). *Chem. Soc. Rev.* **43**, 6097–6115. 10.1039/c4cs00081a 24947910

[bb13] Fathieh, F., Kalmutzki, M. J., Kapustin, E. A., Waller, P. J., Yang, J. & Yaghi, O. M. (2018). *Sci. Adv.* **4**, eaat3198. 10.1126/sciadv.aat3198 PMC5993474 29888332

[bb14] Finsy, V., Kirschhock, C. E. A., Vedts, G., Maes, M., Alaerts, L., De Vos, D. E., Baron, G. V. & Denayer, J. F. M. (2009). *Chem. Eur. J.* **15**, 7724–7731. 10.1002/chem.200802672 19551773

[bb15] Gao, W., Jing, Y., Yang, J., Zhou, Z., Yang, D., Sun, J., Lin, J., Cong, R. & Yang, T. (2014). *Inorg. Chem.* **53**, 2364–2366. 10.1021/ic403175w 24512540

[bb16] Groom, C. R., Bruno, I. J., Lightfoot, M. P. & Ward, S. C. (2016). *Acta Cryst.* B**72**, 171–179. 10.1107/S2052520616003954 PMC4822653 27048719

[bb17] Howarth, A. J., Liu, Y., Li, P., Li, Z., Wang, T. C., Hupp, J. T. & Farha, O. K. (2016). *Nat. Rev. Mater.* **1**, 15018.

[bb18] Krause, L., Herbst-Irmer, R., Sheldrick, G. M. & Stalke, D. (2015). *J. Appl. Cryst.* **48**, 3–10. 10.1107/S1600576714022985 PMC4453166 26089746

[bb19] Ramaswamy, P., Wieme, J., Alvarez, E., Vanduyfhuys, L., Itié, J.-P., Fabry, P., Van Speybroeck, V., Serre, C., Yot, P. G. & Maurin, G. (2017). *J. Mater. Chem. A*, **5**, 11047–11054.

[bb20] Reinsch, H. & De Vos, D. (2014). *Microporous Mesoporous Mater.* **200**, 311–316.

[bb21] Rubio-Martinez, M., Avci-Camur, C., Thornton, A. W., Imaz, I., Maspoch, D. & Hill, M. R. (2017). *Chem. Soc. Rev.* **46**, 3453–3480. 10.1039/c7cs00109f 28530737

[bb22] Schilling, L.-H., Reinsch, H. & Stock, N. (2016). *The Chemistry of Metal–Organic Frameworks: Synthesis, Characterization and Applications*, edited by S. Kaskel, pp. 105–135. Weinheim: Wiley-VCH.

[bb23] Sheldrick, G. M. (2015*a*). *Acta Cryst.* A**71**, 3–8.

[bb34] Sheldrick, G. M. (2015*b*). *Acta Cryst.* C**71**, 3–8.

[bb24] Silva, P., Vilela, S. M. F., Tomé, J. P. C. & Almeida Paz, F. A. (2015). *Chem. Soc. Rev.* **44**, 6774–6803. 10.1039/c5cs00307e 26161830

[bb25] Spek, A. L. (2009). *Acta Cryst.* D**65**, 148–155. 10.1107/S090744490804362X PMC2631630 19171970

[bb26] Stock, N. (2014). *Encyclopedia of Inorganic and Bioinorganic Chemistry*, 2 ed., edited by R. A. Scott, pp. 1–16. Wiley Online Library.

[bb27] Stock, N. & Biswas, S. (2012). *Chem. Rev.* **112**, 933–969. 10.1021/cr200304e 22098087

[bb28] Volkringer, C., Loiseau, T., Guillou, N., Férey, G., Elkaïm, E. & Vimont, A. (2009). *Dalton Trans.* pp. 2241–2249. 10.1039/b817563b 19274304

[bb29] Volkringer, C., Meddouri, M., Loiseau, T., Guillou, N., Marrot, J., Férey, G., Haouas, M., Taulelle, F., Audebrand, N. & Latroche, M. (2008). *Inorg. Chem.* **47**, 11892–11901. 10.1021/ic801624v 19053340

[bb30] Weber, G., Bezverkhyy, I., Bellat, J.-P., Ballandras, A., Ortiz, G., Chaplais, G., Patarin, J., Coudert, F.-X., Fuchs, A. H. & Boutin, A. (2016). *Microporous Mesoporous Mater.* **222**, 145–152.

[bb31] Yuan, S., Feng, L., Wang, K., Pang, J., Bosch, M., Lollar, C., Sun, Y., Qin, J., Yang, X., Zhang, P., Wang, Q., Zou, L., Zhang, Y., Zhang, L., Fang, Y., Li, J. & Zhou, H.-C. (2018). *Adv. Mater.* **30**, 1704303. 10.1002/adma.201704303 29430732

[bb32] Zhang, Y., Lucier, B. E. G., McKenzie, S. M., Arhangelskis, M., Morris, A. J., Friščić, T., Reid, J. W., Terskikh, V. V., Chen, M. & Huang, Y. (2018). *Appl. Mater. Interfaces*, **10**, 28582–28596. 10.1021/acsami.8b08562 30070824

[bb33] Zhou, G., Yang, Y. & Fan, R. (2012). *Inorg. Chem. Commun.* **16**, 17–20.

