# Supplementary material for: One-dimensional ladder gallium coordination polymer
Source: Acta Crystallogr E Crystallogr Commun. 2019 Oct 3;75(Pt 11):1607–12. doi: 10.1107/S2056989019013446 (PMC6829714; doi:10.1107/S2056989019013446)
Supplement: Supplementary file 3 [file e-75-01607-sup3.docx]

**One Dimensional Double Decked Gallium Coordination Polymer**

**Andrea Barres Simões,^1^ Flávio Figueira,^1^ Ricardo F. Mendes,^1^ Jéssica S. Barbosa,^1,2^ João Rocha^1^ and Filipe A. Almeida Paz^1,^***

*^1^Department of Chemistry, CICECO - Aveiro Institute of Materials, University of Aveiro, 3810-193 Aveiro, Portugal*

*^2^QOPNA & LAQV-REQUIMTE, Chemistry Department, University of Aveiro, 3810-193 Aveiro, Portugal*

**Electronic Supporting Information**

**Figure S1.** Powder XRD of [Ga(HPDC)(OH)(H_2_O)] (**I**) prepared by Microwave-Assisted Synthesis (MWAS), Hydrothermal (HT) and One-pot (OP).

**Figure S2.** FTIR spectra of [Ga(HPDC)(OH)(H_2_O)] (**I**) prepared by Microwave-Assisted Synthesis (MWAS), Hydrothermal (HT) and One-pot (OP).

**Figure S3.** Thermogravimetry of [Ga(HPDC)(OH)(H_2_O)] (**I**) prepared by Microwave-Assisted Synthesis (MWAS), Hydrothermal (HT) and One-pot (OP).
